# Clinical Profile, Magnetic Resonance Imaging (MRI) Findings, and Neurological Outcomes in Neonatal Rotavirus Encephalitis: A Prospective Observational Study

**DOI:** 10.7759/cureus.100232

**Published:** 2025-12-28

**Authors:** Vikram Sakaleshpur Kumar, Prashanth S Veeraiah, Gifty Mathew

**Affiliations:** 1 Pediatrics and Child Health, Subbaiah Institute of Medical Sciences, Shivamogga, IND; 2 Pediatric Medicine, Sarji Maternal and Child Hospital, Shivamogga, IND; 3 Pediatrics and Neonatology, Sarji Maternal and Child Hospital, Shivamogga, IND

**Keywords:** encephalitis, mri, neonatal seizures, neurodevelopment, rotavirus

## Abstract

Background: Rotavirus, classically an enteric pathogen, is now recognized as a neurotropic virus capable of causing neonatal encephalitis. Its neurological effects, though rare, have significant developmental consequences.

Objective: This study aimed to evaluate the clinical profile, magnetic resonance imaging (MRI) findings, and neurological outcomes in neonates with rotavirus encephalitis.

Methods: A prospective observational study was conducted in the Neonatal Intensive Care Unit of Sarji Maternal and Child Hospital, Shivamogga, India, from April 2023 to March 2025. Twenty-five neonates aged three to nine days presenting with seizures and encephalopathy were included if MRI suggested viral encephalitis and stool reverse transcriptase polymerase chain reaction (RT-PCR) was positive for rotavirus. Demographic, clinical, biochemical, and radiologic data were prospectively recorded and analyzed using Statistical Package for the Social Sciences version 24 (IBM Corp., Armonk, NY).

Results: Among 25 neonates, 13 (52%) were female and 12 (48%) were male neonates; 20 (80%) were term, and five (20%) were preterm. Nineteen (76%) were outborn, and six (24%) were inborn. Seizure onset occurred less than or equal to four days in 11 (44 %), on day 5 in eight (32%), and after day 5 in six (24%). Common accompanying symptoms were lethargy/poor feeding in 13 (52%), diarrhea or vomiting in eight (32%), and respiratory distress in five (20%). Laboratory findings showed leukocytosis in 15 (60%), thrombocytopenia in 10 (40%), hypocalcemia in five (20%), C-reactive protein positivity in seven (28%), CSF pleocytosis in 12 (48%), and protein elevation in 14 (56%). All 23 tested (100%) had stool RT-PCR-positive for rotavirus. MRI revealed symmetric periventricular±basal ganglia involvement in 14 (56%), superficial periventricular white-matter lesions in eight (32%), and extensive periventricular+deep-gray±brainstem lesions in three (12%). At discharge, 17 (68%) had hypertonia, three (12%) hypotonia, and five (20%) normal tone. At the one-year follow-up, 15 (60%) had normal development, whereas 10 (40%) had abnormal outcomes: global developmental delay in seven (28%), postencephalitic epilepsy in two (8%), and spastic cerebral palsy in one (4%). The severity of MRI abnormalities was strongly associated with adverse neurodevelopmental outcomes (p < 0.001).

Conclusion: Neonatal rotavirus encephalitis manifests with early-onset seizures and distinct MRI signatures that predict later neurological impairment. Early MRI evaluation and structured long-term developmental surveillance are crucial to improving outcomes.

## Introduction

Rotavirus infection remains a leading cause of infant morbidity and mortality worldwide, accounting for nearly 450,000 deaths annually among children under five years [1]. While classically enteric, the neurotropic potential of the virus has become increasingly evident. Case reports and cohort studies describe neonatal encephalopathy with distinctive magnetic resonance imaging (MRI) patterns that implicate involvement of the white matter, basal ganglia, and corpus callosum [2-5].

In neonates, infection is often acquired within the first week of life through nosocomial transmission in nurseries or neonatal intensive care units (NICUs). Clinical manifestations may lack gastrointestinal symptoms; seizures and lethargy can be the sole indicators [3,6]. Rotavirus nonstructural protein 4 (NSP4) acts as an enterotoxin and virotoxin, altering calcium homeostasis and promoting neuronal excitotoxicity [7]. These mechanisms underpin the characteristic imaging features observed in affected infants.

Although Japanese and Korean series have described such “fifth-day fits” with rotavirus-associated leukoencephalopathy [5,8,9], Indian data remain sparse. Understanding clinical and imaging correlations in this setting can guide diagnostic suspicion and follow-up strategies.

This study aimed to characterize the clinical profile, MRI findings, and neurological outcomes of neonates with confirmed rotavirus encephalitis and to assess the relationship between MRI severity and neurodevelopmental outcome.

## Materials and methods

This prospective observational study was carried out in the 27-bedded tertiary-care NICU of Sarji Maternal and Child Hospital, Shivamogga, India, over a two-year period from April 2023 to March 2025. The hospital serves as a regional referral center catering to both inborn and outborn neonates from central Karnataka. During the study period, a total of 1,650 neonates were admitted to the NICU, of whom 180 were diagnosed with encephalopathy based on clinical and neurological assessment. From this cohort, 25 neonates met the predefined inclusion criteria and were enrolled in the study.

Study population and enrollment

Neonates between three and nine days of life who presented with seizures and features of encephalopathy were screened. Eligibility required radiological and virological confirmation: MRI suggestive of viral encephalitis, defined as white-matter hyperintensity or restricted diffusion in periventricular and/or basal ganglia regions; and stool reverse transcriptase polymerase chain reaction (RT-PCR) positive for rotavirus. These two criteria together ensured that only confirmed cases of rotavirus-associated encephalitis were included.

Neonates with major congenital brain malformations, inborn errors of metabolism, hypoxic-ischemic encephalopathy, or bacterial meningitis were excluded to avoid confounding etiologies that could mimic viral brain injury. All included infants were managed according to unit protocols for neonatal seizures, supportive care, and infection control.

Data collection

Detailed demographic, obstetric, and clinical data were prospectively recorded using a structured proforma. Maternal variables included age, parity, antenatal history, and any perinatal complications. Perinatal details, including gestational age, mode of delivery, birth weight, and place of birth (inborn or outborn), were documented. For each neonate, the timing of seizure onset, seizure semiology, associated symptoms (e.g., lethargy, poor feeding, diarrhea, vomiting, or respiratory distress), and relevant systemic findings were documented.

Laboratory investigations comprised complete blood counts, serum electrolytes (including calcium), C-reactive protein (CRP), and cerebrospinal fluid (CSF) analysis for cell count, protein, and glucose levels. Blood cultures were performed to exclude bacterial infection. Stool samples collected within 48 hours of presentation were analyzed for rotavirus RNA using RT-PCR at a certified molecular diagnostics laboratory.

Neuroimaging protocol

MRI was performed on a 1.5-Tesla GE Optima scanner (GE HealthCare Technologies, Inc., Chicago, Illinois). Imaging sequences included T1-weighted, T2-weighted, fluid-attenuated inversion recovery, diffusion-weighted imaging (DWI), and apparent diffusion coefficient (ADC) mapping. Sedation, when required, was administered under neonatologist supervision following standard safety guidelines.

Lesions were categorized based on distribution and depth of involvement into three distinct patterns: 1) extensive: periventricular lesions with extension to deep gray matter and/or brainstem; 2) symmetric periventricular±basal ganglia involvement; and 3) superficial periventricular white-matter lesions limited to subcortical zones.

Radiological interpretation was performed jointly by a pediatric radiologist and the principal investigator to ensure interobserver consistency.

Neurological tone and outcome assessment

Neurological tone at discharge was evaluated using the Simple Neurological Rating Scale (SNRS) [10], a standardized bedside clinical tool that assesses tone, reflexes, and alertness in neonates. This scale is routinely used in neonatal units for neurological screening and does not require formal licensing or external permission. Assessments were performed by trained pediatric residents under consultant supervision to ensure consistency. Follow-up evaluations at three-month intervals and at one year were conducted using the Trivandrum Developmental Screening Chart (TDSC) [11] to document developmental progress and identify early delay.

Statistical analysis

Data were analyzed using IBM Statistical Package for the Social Sciences Statistics version 24.0 (IBM Corp., Armonk, NY). Continuous variables were summarized as means with standard deviations, while categorical data were expressed as frequencies and percentages, n (%). Fisher’s exact test was employed to assess associations between MRI pattern and neurological outcome at one year. A p value of <0.05 was considered statistically significant.

Ethical considerations

The study protocol was reviewed and approved by the Institutional Ethics Committee of Subbaiah Institute of Medical Sciences (IEC-SUIMS/178/2025026). Written informed consent for participation and publication of anonymized data was obtained from the parents or legal guardians of all enrolled neonates. All procedures adhered to the principles of the Declaration of Helsinki.

The SNRS and TDSC used in this study are standard, validated clinical assessment tools routinely applied in neonatal units for neurological and developmental evaluation. As these instruments are in the public domain and require no commercial licensing, no separate permission documentation was necessary.

## Results

A total of 25 neonates met the inclusion criteria during the study period. The demographic and clinical characteristics of the study population are summarized in Table 1. Of these, 13 (52%) were female and 12 (48%) were male neonates. The majority, 20 (80%), were born at term, and 19 (76%) were outborn deliveries referred to the tertiary NICU within the first week of life. Vaginal delivery was the predominant mode, 15 (60%), and most neonates (88%) had a normal birth weight for gestational age.

**Table 1:** Demographic and clinical characteristics of neonates with rotavirus encephalitis (n = 25) Percentages are calculated from the total sample size (n = 25). Each neonate could present with more than one symptom (e.g., lethargy with diarrhea)

Parameter	Category	n (%)
Sex	Female	13 (52)
	Male	12 (48)
Gestation	Term	20 (80)
	Preterm	5 (20)
Place of birth	Inborn	6 (24)
	Outborn	19 (76)
Mode of delivery	Vaginal	15 (60)
	Cesarean section	10 (40)
Seizure onset	≤4 days	11 (44)
	Day 5	8 (32)
	>5 days	6 (24)
Presenting symptoms	Lethargy/poor feeding	13 (52)
	Diarrhea/vomiting	8 (32)
	Respiratory distress	5 (20)

Seizure onset demonstrated a distinctive temporal clustering pattern: 11 (44%) infants developed seizures within the first four days of life, eight (32%) on day 5, and six (24%) after day 5. This early occurrence, often preceding any gastrointestinal symptoms, reflected the classic “fifth-day fits” phenomenon described in neonatal viral encephalopathies. The most common presenting symptoms accompanying seizures were lethargy and poor feeding, 13 (52%), followed by gastrointestinal disturbances such as diarrhea or vomiting, 8 (32%), and respiratory distress, 5 (20%). These findings indicate that gastrointestinal manifestations were not universal, underscoring the neurotropic nature of the infection rather than a purely enteric disease process.

Laboratory and CSF findings

Laboratory evaluation revealed inflammatory or metabolic abnormalities in a subset of neonates (Table 2). CSF pleocytosis was noted in 12 (48%), with mild protein elevation in 14 (56%), while glucose levels remained within the normal range in 20 (80%). Serum analyses showed leukocytosis in 15 (60%), thrombocytopenia in 10 (40%), hypocalcemia in five (20%), and a positive CRP in seven (28%). Bacteremia was detected in six cases (24%), suggesting possible co-infection but not sufficient to explain the neuroimaging pattern. All 23 neonates tested (100%) had stool RT-PCR positive for rotavirus, confirming the diagnosis and supporting a causal link between systemic infection and neurological involvement.

**Table 2:** Laboratory and CSF findings CRP: C-reactive protein; RT-PCR: reverse transcriptase polymerase chain reaction; CSF: cerebrospinal fluid

Parameter	n (%)
Pleocytosis	12 (48)
Protein elevation	14 (56)
Hypocalcemia	5 (20)
Leukocytosis	15 (60)
CRP positive	7 (28)
Thrombocytopenia	10 (40)
Blood culture positive	6 (24)
Stool RT-PCR positive	23 (100)

Magnetic resonance imaging findings

Characteristic MRI abnormalities were observed in all 25 neonates (Table 3). The predominant pattern comprised symmetric periventricular and basal ganglia involvement in 14 (56%), whereas superficial periventricular white matter lesions were seen in eight (32%). The most severe pattern, involving extensive periventricular regions with extension into deep gray matter and the brainstem, was identified in three (12%) neonates.

**Table 3:** MRI pattern and neurological status at discharge MRI: magnetic resonance imaging

MRI pattern	n (%)	Predominant tone at discharge
Extensive (periventricular + deep gray + brainstem)	3 (12)	Hypertonia (3/3; 100%)
Symmetric periventricular ± basal ganglia	14 (56)	Hypertonia (14/14; 100%)
Superficial periventricular white matter	8 (32)	Hypotonia 3 (37.5%), normal 5 (62.5%)

**Figure 1:**
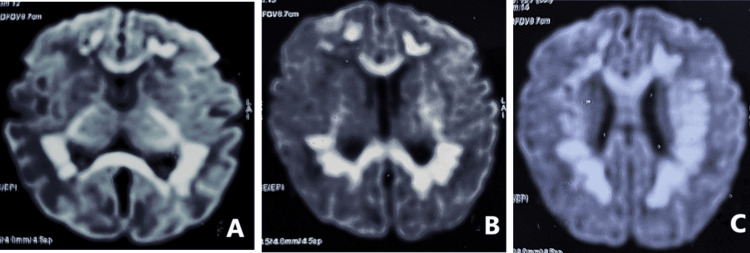
Magnetic resonance imaging demonstrating radiological hallmarks of neonatal rotavirus encephalitis (A) Symmetric diffusion restriction in the periventricular white matter, bilateral internal capsule, and thalami. These lesions appear hyperintense on diffusion-weighted imaging, consistent with cytotoxic edema. (B) Diffusion restriction is seen involving the corpus callosum and bilateral periventricular/deep white matter. (C) Diffusion restriction is seen involving the corpus callosum and bilateral periventricular/deep white matter

Representative MRI images are shown in Figures 1A-1C, demonstrating symmetric diffusion restriction in the periventricular white matter and basal ganglia, radiological hallmarks of neonatal rotavirus encephalitis. These lesions appeared hyperintense on DWI and hypointense on ADC mapping, consistent with cytotoxic edema. The periventricular localization likely reflects the selective vulnerability of metabolically active myelinating regions during early neonatal life.

Neurological status at discharge

Neurological tone at discharge correlated closely with the extent of MRI abnormality. As shown in Table 3, all neonates with extensive or symmetric periventricular lesions exhibited hypertonia, 17 (68%) of the cohort; whereas hypotonia, 3 (12%), or normal tone, 5 (20%), occurred exclusively among those with superficial lesions. These tone abnormalities served as early clinical indicators of subsequent developmental outcomes.

Neurodevelopmental outcome at one year

At one-year follow-up, 15 infants (60%) demonstrated normal developmental milestones, while 10 (40%) exhibited adverse outcomes (Table 4). Among these, seven (28%) had global developmental delay or motor retardation, two (8%) developed postencephalitic epilepsy, and one (4%) was diagnosed with spastic cerebral palsy.

**Table 4:** MRI pattern and one-year outcome MRI: magnetic resonance imaging

MRI pattern	Normal development, n (%)	Abnormal outcome, n (%)	p value
Extensive (periventricular+deep gray+brainstem)	0 (0)	3 (100)	<0.001
Symmetric periventricular±basal ganglia	7 (50)	7 (50)	
Superficial periventricular white matter	8 (100)	0 (0)	

The severity of MRI findings at presentation showed a statistically significant association with neurodevelopmental prognosis (p < 0.001). All three infants with extensive periventricular±brainstem lesions developed persistent neurological deficits, whereas those with isolated superficial periventricular changes had uniformly favorable outcomes. Infants with symmetric periventricular or basal ganglia involvement displayed mixed trajectories, half achieving normal development and half showing varying degrees of delay or tone abnormality.

Overall, the pattern and distribution of MRI lesions emerged as strong predictors of neurodevelopmental outcome, reinforcing the diagnostic and prognostic utility of early MRI evaluation in neonates with unexplained seizures.

## Discussion

This study underscores that rotavirus, traditionally considered an enteric pathogen, can act as a neurotropic virus capable of producing early-onset seizures and characteristic MRI patterns in neonates. The observed clinical constellation, including seizures within the first week of life, absence of perinatal distress, and consistent stool RT-PCR positivity, strongly supports a direct viral etiology rather than hypoxic or metabolic causes. Nearly half of the affected neonates in this cohort experienced seizures within four days of life, resembling the “fifth-day fits” phenomenon described in East Asian and European series of neonatal rotavirus encephalitis [5,8,12].

Significance in the context of existing literature

Rotavirus has been increasingly recognized for its ability to invade the central nervous system (CNS). Evidence from Japan and Korea has established that viremia and hematogenous spread enable the virus to cross the immature blood-brain barrier, causing diffuse white matter and basal ganglia injury [4,6,12,13]. Studies by Lee [4] and Yeom and Park [8] reported symmetric diffusion restriction in periventricular white matter and corpus callosum; patterns identical to those found in our study. Similarly, an Italian multicenter review by Meyer et al. [14] noted that these lesions correlate with motor abnormalities and developmental delay, reinforcing the prognostic value of neuroimaging.

In contrast, earlier Indian studies of neonatal seizures have rarely included viral etiologies in differential diagnosis, focusing instead on hypoxic-ischemic or metabolic causes [12,15,16]. The current findings expand the Indian evidence base by confirming that a substantial subset of early neonatal seizures may have a viral, specifically rotaviral, origin. This has major diagnostic implications for NICUs in low- and middle-income countries where viral panels are not routinely performed.

MRI-outcome correlation and mechanistic insights

The strong association between MRI severity and long-term neurological outcome (p < 0.001) mirrors prior observations that deep gray matter and brainstem involvement are poor prognostic indicators [4,15,17]. The pathophysiological basis is now better understood: rotavirus NSP4 functions as an enterotoxin and virotoxin, altering intracellular calcium homeostasis and disrupting neuronal and oligodendroglial integrity [7]. Experimental models have demonstrated that this calcium imbalance leads to excitotoxic injury and demyelination, findings consistent with the periventricular lesions observed on DWI in our series.

The selective vulnerability of periventricular white matter in early neonatal life likely reflects its high metabolic activity and the rapid myelination occurring in that region. This may explain why superficial periventricular lesions were reversible in some neonates, whereas extensive involvement extending into the thalami and brainstem predicted persistent tone abnormalities and developmental delay.

Comparative perspective

The pattern of outcomes observed in this study, 40% with neurodevelopmental delay or epilepsy, closely parallels the 35%-45% adverse outcome rates reported in Korean [8] and Japanese [15,17] neonatal cohorts. However, unlike those populations where rotavirus vaccination is universal, our cohort represents a largely unvaccinated neonatal population, suggesting that maternal or environmental transmission may play a greater role in India. A recent meta-analysis by Lu et al. [18] reported that neonatal rotavirus infection accounts for up to 12% of acute neonatal encephalitis globally; however, systematic follow-up data from South Asia remain scarce. This study, therefore, fills an important regional evidence gap.

Clinical and public-health implications

These findings highlight the need for heightened clinical suspicion of viral etiologies in early-onset neonatal seizures. Stool RT-PCR for rotavirus should be included in the diagnostic work-up for encephalopathic neonates, even in the absence of gastrointestinal symptoms. MRI serves not only as a diagnostic adjunct but also as a prognostic biomarker, enabling early parental counseling and initiation of neurodevelopmental surveillance.

At the systems level, our results underscore the importance of stringent infection-control practices in NICUs to prevent horizontal transmission. The data also indirectly reinforce the public-health importance of maternal and infant rotavirus immunization, which may reduce community and nosocomial spread.

Limitations and future directions

The study’s single-center design and relatively small sample size limit generalizability. Nevertheless, the prospective design and one-year follow-up provide valuable longitudinal data. Future multicenter studies should incorporate serial MRI and electroencephalographic correlation to map lesion evolution and recovery. Quantification of viral load or inflammatory biomarkers in CSF may also clarify mechanisms linking systemic infection to CNS injury.

In summary, this work adds to the growing evidence that rotavirus is not merely a gastrointestinal pathogen but a cause of neonatal encephalitis with specific imaging and clinical signatures. The study provides one of the first systematic Indian data sets correlating MRI findings with one-year neurological outcomes. By situating rotavirus encephalitis within the broader spectrum of neonatal seizures, these findings invite a paradigm shift in both diagnosis and follow-up of affected infants.

## Conclusions

Rotavirus infection in the neonatal period deserves recognition beyond its gastrointestinal presentation. In its neurological form, it challenges conventional assumptions about the causes of early neonatal seizures. The present study reinforces the importance of looking for viral etiologies in encephalopathic neonates who lack perinatal distress or metabolic triggers.

Early neuroimaging, especially MRI, can reveal a pattern that guides both diagnosis and counseling. More importantly, long-term developmental surveillance should be viewed as a part of essential care, not follow-up optionality. For clinicians, the message is simple but often missed: what seems benign or idiopathic in the first week of life may in fact be the earliest expression of viral brain injury.

Recognition of this entity within neonatal practice; through awareness, timely testing, and structured follow-up; can transform outcomes, reducing lifelong disability and redefining what “routine” neonatal encephalopathy work-ups should include.
